# Different Concentrations of Potassium Silicate in Nutrient Solution Affects Selected Growth Characteristics and Mineral Composition of Barley (*Hordeum vulgare* L.)

**DOI:** 10.3390/plants11111405

**Published:** 2022-05-25

**Authors:** Anja Mavrič Čermelj, Eva Fideršek, Aleksandra Golob, Nina Kacjan Maršić, Katarina Vogel Mikuš, Mateja Germ

**Affiliations:** 1 Biotechnical Faculty, University of Ljubljana, Jamnikarjeva ulica 101, 1000 Ljubljana, Slovenia; eva.fidersek@gmail.com (E.F.); aleksandra.golob@bf.uni-lj.si (A.G.); nina.kacjan.marsic@bf.uni-lj.si (N.K.M.); katarina.vogelmikus@bf.uni-lj.si (K.V.M.); mateja.germ@bf.uni-lj.si (M.G.); 2 Department of Low and Medium Energy Physics, Jožef Stefan Institute, Jamova 39, 1000 Ljubljana, Slovenia

**Keywords:** elemental composition, multielemental stoichiometry, hydroponics, crop plants, Si fertilization

## Abstract

This study was undertaken to determine the effect of potassium silicate (K_2_SiO_3_) on the physiological and growth characteristics and elemental composition of barley plants. Hydroponically grown barley (*Hordeum vulgare* L.) var. Wilma was exposed to four different levels of Si in the form of K_2_SiO_3_ at concentrations of 0 (Si_0_), 0.5 (Si_0.5_), 1 (Si_1_) or 1.5 (Si_1.5_) mM Si. Plants were analyzed for root length, number of dry leaves, number of trichomes, electron transport system activity in mitochondria (ETS), leaf pigment content and elemental composition of roots and leaves. Treatment with Si_0.5_ significantly increased the concentration of total chlorophylls, root length and ETS activity in barley. Plants with no Si added to the nutrient solution had significantly more dry leaves than plants from all Si-treated groups. Necrosis was observed in Si_0_ plants, while leaf damage was not visible in treated plants. According to the results of the study, we evidenced that plants were stressed due to Si deficiency. The addition of K_2_SiO_3_ significantly affected the concentration of Si, K, Ca, Cl, S, Mn, Fe and Zn in roots and leaves of barley. In barley treated with Si_0.5_, plants showed the best performance in terms of their physiological characteristics and growth.

## 1. Introduction

Silicon (Si) is an abundant element in the environment and composes 28.8% of the continental crust [1]. It is present in different forms in the soil and can be found in a liquid or an amorphous or crystalline solid phase. The liquid phase is composed of monosilicic, polysilicic acid or complexes of silicic acid and inorganic compounds [2]. Silicic acid can also form complexes with organic compounds [3]. Although various Si compounds are available, plants gain Si from soil water only in the form of monosilicic acid (H_4_SiO_4_) [4].

The means of entry of Si into the cell through pores depends on the plant species. The concentration of Si in plant tissues varies in different plant species and is between 0.1 and 10% of dry weight of the plant [5]. A variation in Si content between varieties within a species has been found. In grains of different barley varieties, the Si concentration can vary from 0 to 0.36% [6]. The Si concentration in barley has been strongly correlated with the expression level of HvLSi2 which controls Si uptake in plants [7]. Plants are divided into three groups according to the Si content in dry shoot weight and the Si:Ca ratio. Si accumulators (>1% Si, Si:Ca > 1) are monocotyledons, e.g., barley and some pteridophytes and bryophytes have been encountered. Intermediate types (0.5–1% Si, Si:Ca = 0.5–1) include some dicotyledons such as cucurbits and nettles. A third group consists of Si excluders (<0.5% Si, Si:Ca < 0.5) and contains other dicotyledons [8].

Silicon is known to mitigate stress caused by different biotic and abiotic stress factors, such as salinity, high temperatures, high UV radiation, drought, heavy metal toxicity, pests, diseases or herbivory. Under high salinity, Si can increase levels of K and suppress Na uptake [9]. Si can increase heavy metal tolerance with structural changes in plants, regulation of the transport genes expression, chelation, compartmentation, stimulating antioxidants, reducing metal transport to the shoots or reducing active metal ions in growth media [10]. There have been many studies on the positive effects of Si on plants under biotic or abiotic stress, but fertilization with Si is not used widely in agriculture [11].

Some studies reported that Si has little or no beneficial effect on plant physiology in stress-free conditions [12], but subsequent research has revealed improved plant performance as well as plant nutrition in non-stressed plants [13]. Si can increase Si accumulation in shoots and grain production in soybean and rice [14]. In strawberries, Si improves vegetative growth, fruit dry weight and significantly modifies the profile of phenolic compounds and activity of metabolic enzymes, resulting in improved quantity and quality parameters of fruits [15]. Si can mediate the uptake and translocation of several nutrients [16]. It decreases the concentration of C and increases the level of P in wheat (*Triticum aestivum* L.) [17]. It decreases the level of Ca, Mn, Fe, Co, Cu and Zn in leaves of common reed (*Phragmites australis* (Cav.) Trin. ex Steud.) [18]. A decrease in Zn caused by Si was found in maize shoots and roots exposed to high concentrations of Zn, suggesting the antagonistic effect of Si [19]. Silicon in unstressed Arabidopsis (*Arabidopsis thaliana* (L.) Heynh.) var. Columbia and sorghum (*Sorghum bicolor* (L.) Moench) increases biosynthesis of phytohormone cytokinin, and this promotes cell division and slows down the senescence process [20]. The addition of Si also delayed senescence in the leaves of rice (*Oryza sativa* L.) [21].

To date, most studies have been focused on the effect of Si addition on the performance of plants and information about the effect of Si deficiency in plants is limited. Deficiency of Si might play an important role in plants such as grasses, that accumulate high concentrations of Si. Seven of the ten most-abundant crops in the world are Si accumulators, and their Si tissue content is >1% [22]. These include barley, which is one of the oldest crops, and is grown for multiple purposes. The limit of information on the effects of Si deficiency is partly due to the fact that Si is a very common element in the earth’s crust and support of conditions that include a severe lack of Si to perform experiments is difficult. However, many years of removal of bioavailable Si from the soil with intensive cultivation of monocultures in specific locations will cause Si deficiency and future crops will probably require application of additional Si to maintain maximum yields [23]. In the present study, we compared untreated barley plants, with plants treated with different concentrations of Si. The Si was added to a nutrient solution in the form of K_2_SiO_3_. The aim of the study was to find the effect of Si deficiency on barley performance and the threshold of Si concentration that induces the physiological, biochemical and morphological response of plants. Special attention was paid to determine, how the increasing concentration of Si in the nutrient solution affects the Si concentration and the elemental composition of barley root and shoots. We hypothesized that (i) since barley is a monocot, Si deficiency would negatively affect plant performance at a certain stage of their development and (ii) Si would affect the accumulation of elements in the roots and leaves of barley.

## 2. Results

### 2.1. Morphological, Biochemical and Physiological Characteristics of Barley Plants

**Figure 1:**
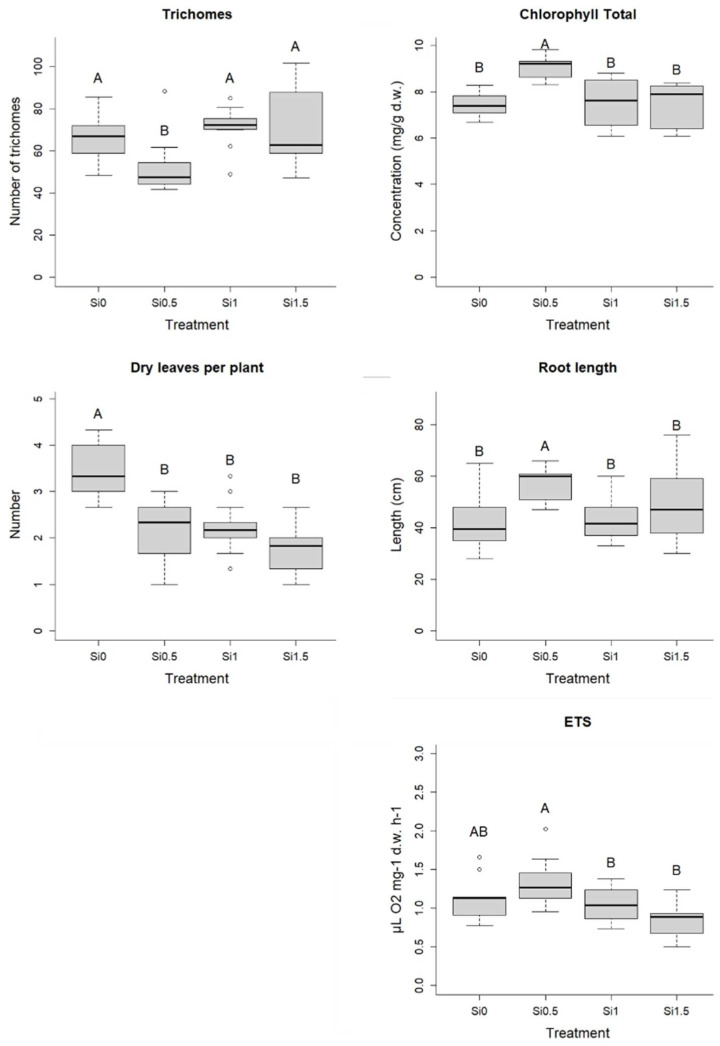
Number of trichomes, number of dry leaves per plant, total chlorophyll content (mg/g d.w.), root length (cm) and ETS activity (µL O_2_ mg^−1^ d.w. h^−1^) for different Si treatments (Si_0_, Si_0.5_, Si_1_, Si_1.5_). Different letters represent statistically significant differences between treatments (one-way ANOVA (Duncan) or Kruskal–Wallis (Dunn), *p* < 0.05, N = 9–18).

Barley leaves after treatment with Si_0.5_ had a lower number of trichomes on their upper and lower epidermis compared to leaves of plants from other treatments. Treatment with Si_0.5_ increased the concentration of total chlorophylls (a sum of chlorophyll A and chlorophyll B), root length and ETS activity in barley when compared to other treatments. The number of dry leaves per plant was the highest in control plants (Si_0_), indicating increased senescence. Plants treated with Si_0.5_ had longer roots compared to plants from other treatments (Figure 1).

We found no statistically significant differences between treatments in the effective photochemical efficiency of PS II (Y). The potential photochemical efficiency of PS II (Fv/Fm) ranged from 0.75 to 0.81 during the experiment, with an average of 0.80, indicating that the plants were in good condition (data not shown).

**Figure 2:**
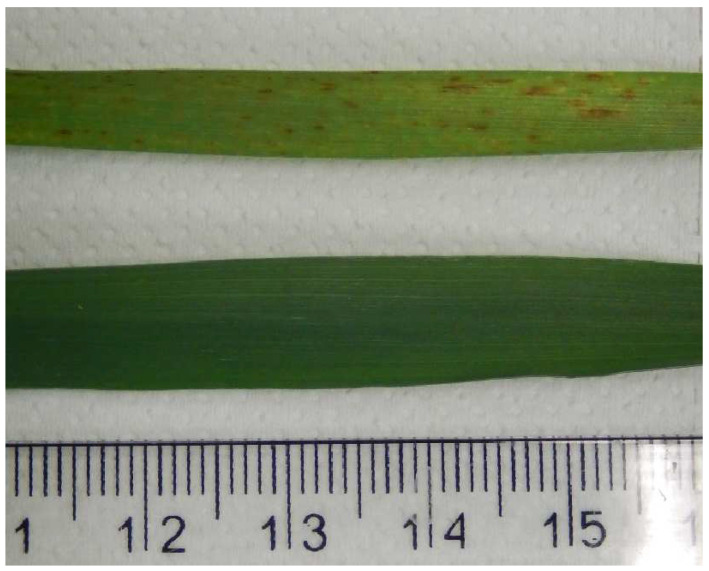
Comparison between leaves from different treatments with visible necrosis. Upper leaf: Si_0_, bottom leaf Si_0.5_. Scale in cm.

After 32 days of treatment, the first necrosis was observed on leaves in Si_0_ treatment (Figure 2). Necrosis was present in all Si_0_-treated plants, and the most severe damage was present on older leaves. No necrosis on barley leaves was observed in treatments with added K_2_SiO_3_.

### 2.2. Elemental Composition

**Figure 3:**
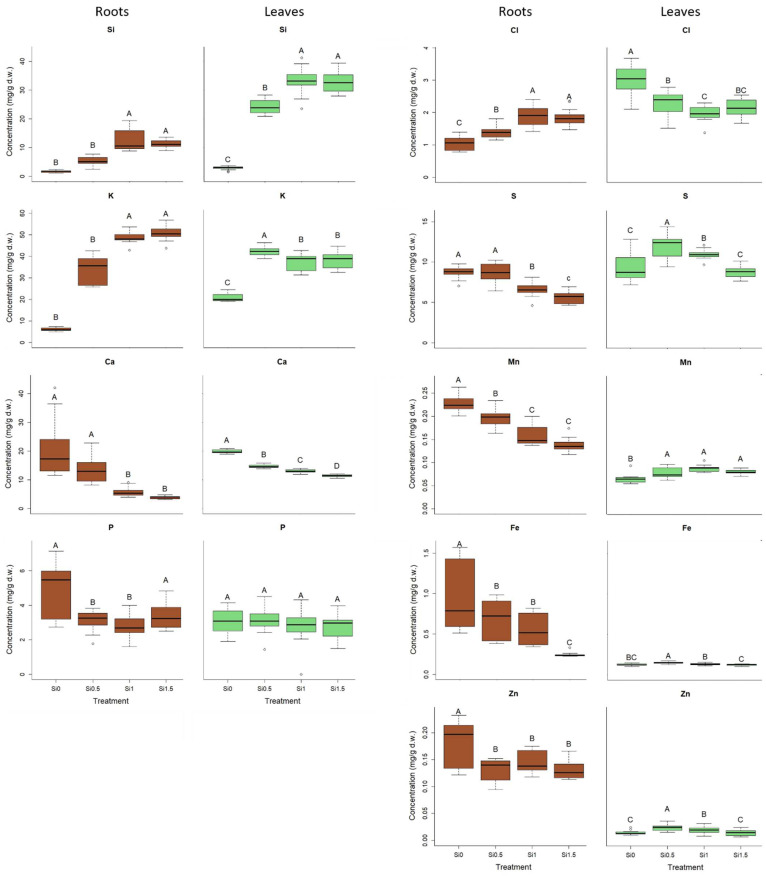
Concentration of Si, K, Ca, P, Cl, S, Mn, Fe and Zn (ppm) in roots and leaves for different Si treatments (Si_0_, Si_0.5_, Si_1_, Si_1.5_). Different letters represent statistically significant differences between treatments (one-way ANOVA (Duncan) or Kruskal–Wallis (Dunn), *p* < 0.05, N = 9–15).

An increased concentration of added Si in the nutrient solution increased the Si concentration in the roots and leaves of barley (Figure 3), but the concentration of Si did not differ between plants treated with 1 (Si_1_) and 1.5 (Si_1.5_) mM Si (Figure 3). The Si concentration was higher in the leaves than in the roots in each treated plant. The concentration of K in roots and leaves increased with the increasing concentration of K_2_SiO_3_ in the nutrient solution. With increasing Si concentration in the nutrient solution, the content of Ca decreased in barley roots and leaves. A similar result was noted in the concentration of Mn, S, Fe and Zn in the roots. The concentration of Mn in leaves was higher after treatment with Si_0.5_, Si_1_ and Si_1.5_ rather than with Si_0_. The concentration of S was the highest in the Si_0.5_-treated plants, and this was also true of the concentration of Fe and Zn in leaves. Regardless of Si treatment, the concentration of Mn, Fe and Zn was higher in roots than in leaves. The concentration of Cl in roots increased with increasing Si in the nutrient solution, but in contrast, the concentration of Cl in the leaves decreased as the concentration of Si in the nutrient solution increased (Figure 3).

**Figure 4:**
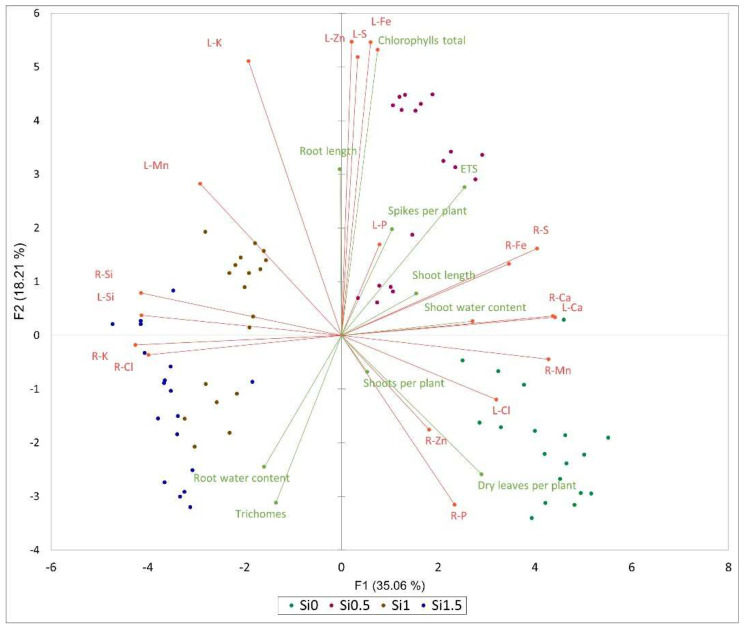
PCA analysis of some morphological and biochemical data, and elemental composition of roots and leaves. Abbreviations: R—roots, L—leaves.

Principal component analysis (PCA) revealed correlations between the different parameters (Figure 4). Negative correlations were observed between the content of Si and Ca in roots and leaves. The Si concentration was also negatively related to the concentration of Mn in roots (L-Si:R-Mn was −0.799, and R-Si:R-Mn was −0.748; *p* ≤ 0.05), but not in leaves (L-Si:L-Mn was −0.154). The Si concentration in leaves was strongly positively correlated with the Cl concentration in roots (0.814, *p* ≤ 0.05), but strongly negatively correlated to the Cl concentration in leaves (−0.629, *p* ≤ 0.05). The Si concentration in roots was strongly positively correlated with the K concentration in roots (0.849, *p* ≤ 0.05), but the correlation of Si in roots with the K concentration in leaves was weak (0.425, *p* ≤ 0.05).

The number of dry leaves per plant was negatively correlated with K in leaves (−0.665, *p* ≤ 0.05) with the Si in roots (−0.622, *p* ≤ 0.05) and leaves (−0.543, *p* ≤ 0.05). The concentrations of Ca and S in roots were positive correlated (0.601 and 0.620, respectively, *p* ≤ 0.05) with the ETS activity in barley leaves.

## 3. Discussion

### 3.1. Morphological, Biochemical and Physiological Characteristics of Barley Plants

Numerous studies have shown the positive effects of Si addition to the nutrients in many plant species, especially when exposed to conditions of stress [12,24]. Recent studies have shown that the addition of Si also has a positive effect on non-stressed plants [14,15,17]. There is however a lack of data on the impact of Si deficiency on plant growth and development, which could be important, particularly in species that are Si accumulators. The results of our experiments show that plants with no Si added to the nutrient solution—Si_0_—had a significantly higher number of dry leaves at the end of the experiment than plants from all Si-treated groups. In Si_0_ plants, we also observed necrosis, which emerged in the late flowering phase of the plant development only in Si_0_-treated plants. On the other hand, leaf damage was not visible in treated leaves. The barley with no Si added had a significantly lower content of total chlorophyll than barley from Si_0.5_ treatment but not from Si_1_ and Si_1.5_ treatments. The first visible signs of impaired barley performance in the Si_0_ group were observed in the last 14 days of the experiment, as flowering is beginning. Prior to the flowering phase, plants did not differ between treatments in the measured biochemical and morphological properties and were in a good physiological condition with a potential photochemical efficiency of photosystem II (Fv/Fm) = ~0.8 (data not shown). These results indicate very early senescence in Si_0_-treated barley which could be a consequence of Si deficiency, occurring in certain phases of the plant’s development. Hastened senescence, which occurred in Si_0_ barley, is not desirable as it could reduce crop yield [25]. Si deficiency could also be responsible for the imbalance between minerals in barley root and leaf tissue as discussed below. It has been reported before that symptoms of Si deficiency include declining shoot dry weight, reduced yields, necrosis and chlorosis and wilting of leaves, increased susceptibility to various diseases, disturbances in pollination and fruit development, and accelerated senescence [26]. In the present study, symptoms usually first manifested on older leaves and eventually progressed to younger leaves. It is also possible however that rapid senescence is also a consequence of K deficiency or the combined K and Si deficiencies, but the basic nutrient solution was prepared for barley according to Podar (2013) [27], and K deficiency is not expected. It has been shown that the signaling responses of K deficiency are linked with ethylene synthesis [28], which might induce senescence in plants [29]. Previous studies reported that Si fertilization increased the number and size of solid amorphous silica bodies such as trichomes and prickle cells [30]. Some studies revealed that Si in prickle cells influenced leaf reflectance of light [31,32].

Treatment with Si_0.5_ increased the concentration of total chlorophylls, root length and the electron transport system activity in barley. ETS activity could have been increased because of increased energy demand for the synthesis of pigments and for growth. When organisms demand more energy, ATP production and O_2_ consumption in mitochondria increase [33,34]. No direct studies have been published however on the relationship between respiratory potential and Si content in a growth medium. Based on our results, the nutrition solution with 0.5 M K_2_SiO_3_ induced positive physiological and growth responses and the best performance of barley plants.

### 3.2. Elemental Composition

Results from this research revealed a significant impact of K_2_SiO_3_ on the concentration of Si in barley plants. Growth in a nutrient solution with added K_2_SiO_3_ also significantly affected the concentrations in plants of other elements. Similar to other monocotyledons, barley is known to be a Si accumulator which contains genes that encode Si influx transporters (HvLsi1, HvLsi6) and an efflux transporter (HvLsi2) [35]. The LSi6 transporter is a key factor which enables transport of Si in the form of silicic acid from roots to leaves. In our case, the concentration of Si in leaves is higher than in roots, probably due to Si precipitation in phytolhyths [36]. Higher concentrations of Si in shoots rather than in roots were also found in wheat treated with Si [37]. Our results confirm the accumulation ability of Si in tissues of barley. With higher concentrations of Si in the nutrient solution, the Si concentration in roots and leaves increased. The Si increase in tissues continues up to a certain concentration. In treatments with Si_1_ and Si_1.5_, the Si concentration in the nutrient solution was excessive, and probably led to the precipitation of Si. Precipitation may have also been induced by Ca, which would explain the negative correlation of the Si concentration in plant tissues with the Ca concentration in leaves and roots. Decreased Ca accumulation at increased Si concentrations has been explained by the ability of Si to induce lower transpiration [38], and decrease in transpiration consequently reduces the Ca passive uptake [39]. The decrease in Ca concentration in plants is also explained by the reduced Ca^2+^ uptake as a result of biosilicification of root structures [40] or Si–Ca interaction in the growing media or apoplast [41].

With the increasing concentration of Si in the nutrient solution, the concentration of K in shoots and roots increases. This could be partly because upon the addition of K_2_SiO_3_ to the nutrient solution, the concentration of both Si and K increases. However, significantly lower K concentrations in the roots and leaves of Si_0_-treated plants compared to Si_0.5_-treated plants and other treatments could indicate that Si in nutrient solution stimulates the intake of K in barley. There is a strong correlation between Si and K concentrations in barley roots, but the increase in K concentration in roots was highest in the Si_0_ and Si_0.5_ treatments. With higher concentrations of K_2_SiO_3_ in the nutrient solution, the increase in K uptake in roots was diminished, indicating that the maximal uptake of K was probably achieved upon addition of 1.5 mM K_2_SiO_3_ (Si_1.5_). Yan et al. [42] reported the presence of an Si up-regulating K+ transporter gene, OsHAK5, OsAKT1 and OsSKOR. Increased K uptake by rice plants that had been supplied with Si and were grown under saline conditions may possibly be explained by Si-mediated stimulation of H^+^-ATPase activity [43]. It was reported earlier, in a study of salt-stressed barley, that Si addition can improve the uptake of K by the root and raise the K concentration in both shoot and root [44].

The addition of Si to growth media lowered the concentration of Fe in the roots decisively but had a lesser effect on Fe concentration in leaves. The concentration of Fe in barley roots decreased with increasing concentration of Si in the growth medium. The addition of Si to the growth medium may have caused higher Fe plaque formation that decreased Fe uptake and activation of root Fe deficiency responses as has been reported in rice [45] and cucumber [46]. That occurred even with an optimal Fe supply, supporting the hypothesis by Coskun et al. [47] that Si might cause an apoplastic obstruction.

Similar to Fe, we observed that increased concentration of Si in growth medium significantly lowered the accumulation of Mn in barley roots. The opposite pattern was observed for leaves. In treatment with Si_0_, the concentration of Mn in roots was the highest but the redistribution to leaves was lower than after other treatments. In contrast to our results, Greger et al. (2018) [13] reported that Si promotes Mn accumulation in plants, but more in shoot than in roots. The authors concluded that Si promotes Mn translocation more to the shoot than the uptake of Mn by the plant roots in various plant species grown under conditions of adequate Mn supply.

Silicon in the nutrient solution increased the Cl concentration in barley roots and decreased the concentration of Cl in the leaves. It has been reported that Si decreases the Cl concentration in the shoots of tomato and spinach [48], the root of grapevine [49] and the root, stem and leaves of aloe [50]. However, all experiments were conducted on plants exposed to different kind of stress, and this differs from our experiments. The mechanism by which Si affects Cl transport and its distribution in plant tissues awaits elucidation [16].

Concentrations of Si higher than 0.5 mM in the nutrient solution decreased the concentration of S in barley roots than the Si_0_ and Si_0.5_ treatments. Buchelt et al. (2020) [51] discovered that uptake and accumulation of S was unaffected by the Si supply in the forage crops BRS Zuri (*Panicum maximum* Jacq.) and BRS RB331 Ipyporã (*Brachiaria ruziziensis* × *Brachiaria brizantha*). Si supply decreased shoot accumulation of S in barley and rice [52,53]. We observed similar results in the present study, but only in cases where higher amounts of added Si decreased S concentration in leaves.

## 4. Materials and Methods

Seeds of barley (*Hordeum vulgare* L.) var. Wilma were sterilized for 15 min in 3% sodium hypochlorite and washed well in distilled water. Sterilized seeds were transferred to moist paper towels for germination at room temperature. After five days, the seedlings were transferred to plastic hydroponic net pots and stabilized with foam. The seedlings were grown in for barley modified nutrient solution containing (in mM): 0.4 KH_2_PO_4_, 0.4 K_2_SO_4_, 0.6 MgSO_4_ × H_2_O, 1 NH_4_NO_3_, 2 Ca(NO_3_)_2_, 0.0016 H_3_BO_3_, 0.0036 Mn chelate, 0.003 ZnSO_4_ × 7H_2_O, 0.0008 CuSO_4_ × 5H_2_O, 0.00083 Na_2_Mo × 2H_2_O and 0.075 Fe-chelate [27]. After 15 days of acclimation in nutrient solution, plants were transferred to a floating hydroponic system in 30 L tanks with nutrient solution. The nutrient solution was aerated for 30 min every 4 h. The pH was adjusted and checked every two days and retained at 5.5–6 with either 70% (13 M) H_2_SO_4_ or 1 M KOH. Plants were grown in a greenhouse at 15 °C during the day and 10 °C at night under light panels with a 16/8 light regime (average radiation 300 µmol/m^2^ s). Plants were exposed to four different treatments with 0 (Si_0_), 0.5 (Si_0.5_), 1 (Si_1_) or 1.5 (Si_1.5_) mM Si, which was added to the nutrient solution in the form of K_2_SiO_3_ (Growth Technology, Norton Fitzwarren, UK). The nutrient solution was added when plants used up approximately 10 L of it, and it was completely changed after 33 days of the experiment. Three replications of each treatment were conducted. Each replication contained 24 net pots with three plants in each pot. Every week, the photochemical efficiency of the plants was measured on five different plants from each replication. After 53 days of treatment, plants were sampled for analysis. Plants from six randomly chosen pots per replication were analyzed for root length, the number of dry leaves and trichomes, electron transport system (ETS) activity in mitochondria, leaf pigment content and elemental composition were determined.

### 4.1. Morphological and Anatomical Properties

The number of trichomes on the upper and lower epidermis was determined under 10× magnification under a microscope (CX41; Olympus; Tokyo, Japan), equipped with a digital camera (SC180; Olympus; Tokyo, Japan) using a computer program, CellSens Entry. Trichomes were counted on five different sampling frames from the left to the right side of the leaf. The size of each sampling frame was 600 × 600 µm. Trichomes from three first fully developed leaves per replication were counted. To avoid double-counting, we counted trichomes that were located inside, on the top and on the right side of the sampling frame. Trichomes from upper and lower epidermis were summed for statistical analysis.

Root length was measured from the top of the root to the end of the longest root.

### 4.2. Physiological Analysis

Chlorophyll fluorescence was monitored every week during plant growth on a hydroponics system with five randomly chosen plants per replication. Chlorophyll fluorescence was measured with a portable chlorophyll fluorometer (PAM-2500; Walz, Germany). The potential (Fv/Fm) and effective (Y) photochemical efficiency of photosystem (PS) II were evaluated according to Schreiber et al. (1996) [54].

### 4.3. Biochemical Analysis

Chlorophyll a, chlorophyll b and carotenoid contents were determined by Monni’s method [55] with some modifications. Freeze-dried and milled leaf material (30 mg) was weighed into centrifuge tubes, and 80% acetone (17 mL) was added. Covered centrifuge tubes stored left overnight in the refrigerator (4 °C). The following day, samples were centrifuged for 3 min at 2500 rpm. The absorbance was measured at 647, 664 and 470 nm using a UV/VIS spectrometer (Lambda 25; Perkin-Elmer, Norwalk, CT, USA). Results were expressed as mg of pigment per gram of dry weight. The total content of chlorophyll a, chlorophyll b and carotenoids was used for statistical analysis.

Electron transport system activity in mitochondria (ETS) was measured according to the protocol created by Packard 1971 [56]. A sample of a known area of fresh first fully developed leaf was homogenized in a grinder with 4 mL of ice-cold homogenization buffer at pH 8.4, then transferred to test tubes and ultrasonically homogenized for 20 s. The homogenates were centrifuged at 10,000 rpm and 0 °C for 4 min (Sigma 2-16PK, Sigma Zentrifugen, Osterode am Harz, Germany). Supernatant in triplicate, (0.5 mL) was incubated in 1.5 mL of substrate solution with 0.5 mL INT for 40 min at 20 °C in the dark. After incubation, 0.5 mL stopping solution (H_3_PO_4_:HCHO = 1:1) was added and the absorbance was determined at 490 nm with a UV/VIS spectrophotometer (Lambda 25; Perkin-Elmer, Norwalk, CT, USA). The content of oxygen was determined as described by Kenner (1975) [57].

### 4.4. Elemental Composition

For the purpose of elemental analysis, roots and leaves were washed with deionized water, frozen and freeze-dried. The dried material was milled, and 400 mg pellets were made. The elemental composition of samples was measured with an X-ray fluorescence spectrometer [58] with Fe-55 and Cd-109 source (Eckert & Ziegler, Berlin, Germany).

### 4.5. Statistical Analysis

The statistical software XL Stat for Excel (Version 2112, Addinsoft, Paris, France) was used for statistical analysis. Normal distribution of data was checked with Shapiro–Wilk tests (*p* ≤ 0.05). Upon confirmation of normal distribution, data was further analyzed with one-way analysis of variance (ANOVA) with Duncan’s post hoc tests. If data failed to follow a normal distribution, the non-parametric test Kruskal–Wallis and multiple pairwise comparisons using Dunn’s procedure were used to evaluate differences between the treatments. PCA analysis was accomplished with XL Stat for Excel.

## 5. Conclusions

Hydroponically grown barley was exposed to different levels of K_2_SiO_3_. Increased ETS activity in Si_0.5_-treated barley showed the highest metabolic activity of barley. The plants showed the best performance at this nutrient concentration. Si and/or K deficiency was observed in plants with no K_2_SiO_3_ added to the nutrient solution. These untreated plants had significantly more dry leaves than plants from all Si-treated groups. Necrosis developed exclusively in untreated plants. Treatment with K_2_SiO_3_ significantly affected the concentration of Si, K, Ca, Cl, S, Mn, Fe and Zn in barley roots and leaves.

## Data Availability

Data are available from the authors on request.
